# Multi-Metal Leachate from Lithium Slag Induces Oxidative Stress, Circadian Disruption, and Neurobehavioural Toxicity in Zebrafish Larvae

**DOI:** 10.3390/toxics14040345

**Published:** 2026-04-20

**Authors:** Xueping Huang, Shengping Zhang, Yu Liu, Shuai Liu, Qiyu Wang, Nannan Wan, Shanghaojun Lu, Yongming Wu, Miao Zhang

**Affiliations:** 1 School of Civil Engineering and Architectural Engineering, Jiangxi University of Water Resources and Electric Power, Nanchang 330099, China; hxp_2002@126.com (X.H.); zhangshengping1218@163.com (S.Z.); 13970793227@163.com (S.L.); 2 Institute of Resources and Environment, Jiangxi Academy of Sciences, Nanchang 330096, China; liuyu@jxas.ac.cn (Y.L.); wannannan@jxas.ac.cn (N.W.); 3 Institute of Medicine and Health, Jiangxi Academy of Sciences, Nanchang 330096, China; liushuai@jxas.ac.cn (S.L.); ihbwangqiyu@126.com (Q.W.)

**Keywords:** lithium slag leachate, zebrafish, neurotoxicity, circadian rhythm

## Abstract

The rising global demand for lithium has led to substantial accumulation of lithium slag, a by-product of lithium carbonate production and a potential environmental contaminant. Leachates from this material contain various metal elements and may pose risks to ecosystems and organismal health. However, research on its neurotoxicity and underlying mechanisms remains limited. In this study, zebrafish embryos at 6 h post-fertilisation were exposed to varying concentrations of lithium slag leachate for 7 days. The leachate contained multiple metal ions (Li, Fe, Mn, Ni, Zn, As, Cr, Cu, Hg, Cd, Pb, etc.). Following exposure, significant metal accumulation was observed in larvae, accompanied by developmental malformations (yolk sac oedema, cardiac haemorrhage, and uninflated swim bladders). Behavioural assessment revealed reduced swimming distance and velocity, along with disrupted circadian rhythms. Biochemical analyses showed elevated Reactive oxygen species (ROS), Superoxide dismutase (SOD), Catalase (CAT), and Malondialdehyde (MDA), alongside decreased Glutathione (GSH), indicating oxidative stress. Transcriptomic analysis confirmed downregulation of core circadian genes. Neurotransmitter assays revealed decreased acetylcholine (Ach), noradrenaline (NE), and dopamine (DA), with increased gamma-aminobutyric acid (GABA) and serotonin (5-HT). These findings demonstrate that lithium slag leachate induces oxidative stress, circadian disruption, and neurobehavioural toxicity in zebrafish, providing important evidence for environmental risk assessment.

## 1. Introduction

In recent years, the global energy crisis has intensified because of the overexploitation and continued reliance on conventional fossil fuels such as coal, oil, and natural gas. Lithium-based batteries, which form the cornerstone of modern energy storage technologies, play a critical role in advancing renewable energy systems [1]. Owing to its unique electrochemical properties, lithium is widely used across multiple sectors, including manufacturing (e.g., glass, ceramics, lubricants, and polymers), healthcare, and nuclear technology (e.g., tritium production) [2]. Industrial lithium products primarily include lithium carbonate, lithium hydroxide, and lithium chloride. Among these, lithium carbonate is a key raw material for electric vehicle batteries, and its production and consumption have increased steadily in recent years [3]. The industrial production of lithium carbonate typically uses α-spodumene as the raw material. After mechanical crushing and high-temperature calcination, α-spodumene is converted into the more reactive β-spodumene, which is then reacted with concentrated sulphuric acid to produce soluble lithium sulphate. Subsequent processing steps—including leaching, neutralisation, and purification—precede the addition of sodium carbonate to precipitate the final lithium carbonate product [4]. However, this process generates substantial solid waste, with the mass ratio of lithium residue to lithium carbonate reaching as high as 9:1 [5,6]. Lithium slag, a by-product of lithium product processing, exhibits a glassy structure, a high specific surface area, and strong adsorption capacity [7]. Globally, approximately 1.2 million tonnes of lithium slag are generated annually, of which less than 10% is recycled for building materials such as concrete, while the remainder is disposed of as solid waste [8]. In China alone, annual lithium slag production exceeds 240,000 tonnes, mainly concentrated in provinces such as Jiangxi and Xinjiang [9]. Unlike conventional industrial solid wastes (e.g., blast furnace slag and fly ash), which have relatively stable compositions, lithium slag is chemically heterogeneous. It contains elevated levels of alkali metals (potassium and sodium), sulphur content ranging from 5% to 30%, and residual traces of various metal ions [10]. For instance, lithium mica slag contains potentially hazardous metals derived from feldspar minerals, such as manganese, nickel, and beryllium [11,12]. Current disposal methods, primarily landfilling and impoundment, not only consume land resources but also pose potential ecological risks [13].

If not properly managed, multiple metallic elements present in lithium slag can leach into aquatic and soil environments, posing threats to downstream ecosystems and aquatic organisms. Excessive accumulation of heavy metals in fish can lead to various toxic effects, including carcinogenicity, teratogenicity, and mutagenicity. In particular, metals are known to damage the nervous system of fish, disrupting their behavioural responses and potentially affecting ecological balance [14]. For example, Billah et al. used atomic absorption spectroscopy to determine arsenic and lead levels in dried fish species (silver carp, spotted snakehead, and Chinese bream) and found that spotted snakehead exhibited the highest concentrations of arsenic and lead. Extracts from these species induced developmental toxicity in zebrafish embryos, including abnormal cleavage, pericardial oedema, and spinal curvature [15]. Liu et al. reported that combined exposure to lead (4 mg/L) and arsenic (3.2 mg/L) disrupted the cholinergic system, dopamine (DA) and serotonin (5-HT) signalling, hypothalamic–pituitary–adrenal axis function, and neurodevelopmental gene expression in zebrafish, ultimately exacerbating neurotoxicity and causing abnormal swimming behaviour [16]. Manganese exposure has also been shown to induce apoptosis of dopaminergic neurones and reduce tyrosine hydroxylase expression in zebrafish larvae, resulting in motor deficits and developmental impairment [17]. Exposure of parent zebrafish to cadmium chloride leads to an imbalance in neurotransmitters in the F1 offspring, which in turn causes motor dysfunction [18].

Interestingly, although lithium is a well-established mood stabiliser used to treat bipolar disorder by reducing manic episodes, maintaining long-term stability, and lowering suicide risk, studies have shown that lithium chloride may affect signalling pathways and immune responses, thereby inducing circadian rhythm disturbances [19,20]. The circadian clock is regulated by a transcription–translation feedback loop, in which the Clock gene and its partner BMAL1 activate the expression of repressors such as Per and Cry, which in turn suppress BMAL1 expression, forming an approximately 24 h cycle [21,22]. Research indicates that the light-induced genes zPer2, zCry1a and zCry2a are involved in the regulation of oxidative stress [23]. To date, research on the ecological risks of lithium slag leachate remains limited, particularly with respect to its neurotoxic effects on aquatic organisms, highlighting the need for further investigation.

Zebrafish serve as a valuable vertebrate model in toxicological studies because of their short life cycle (reaching sexual maturity within 3 months), small size, low experimental cost, rapid embryonic development, and optical transparency, which enables direct microscopic observation of developmental stages [24,25]. Furthermore, their central nervous system shares considerable structural and functional homology with that of mammals. Consequently, assessing the neurotoxicity of pollutants using zebrafish can help elucidate their pathogenic molecular mechanisms and provide a theoretical basis for environmental monitoring, early warning systems, and disease prevention strategies. In this study, zebrafish were employed to assess the developmental, neurological, and behavioural effects of exposure to lithium slag leachate at various concentrations. By systematically analysing its toxic impact on the zebrafish nervous system, this study aims to provide toxicological evidence to support the ecological risk assessment and management of lithium slag.

## 2. Materials and Methods

### 2.1. Preparation and Composition Analysis of Lithium Slag Leachate

The lithium slag used in this study was prepared from an industrial source. Fresh slag samples were collected, air-dried, and homogenised. Leaching extraction was performed according to the “Solid waste-Extraction procedure for leaching toxicity-Sulphuric acid & nitric acid method” (HJ/T299-2007) [26]. Preparation of extraction reagent: Add a mixture of concentrated sulphuric acid and concentrated nitric acid in a mass ratio of 2:1 to reagent water (approximately 2 drops of the mixture per litre of water), adjusting the pH to 3.20 ± 0.05. Briefly, lithium slag samples were dried at 105 °C to constant weight, and the moisture content was determined to be 22.18%. A 150 g aliquot of the dried slag was transferred to a 2 L extraction bottle, to which 1.5 L or 0.15 L of extraction solvent was added, corresponding to solid-to-liquid ratios of 1:10 and 1:1 (kg/L), respectively. The sealed bottles were agitated on an inverted shaker at 30 rpm for 18 h at 23 ± 2 °C. The resulting leachate was collected by vacuum filtration using a pressure filter assembly, passed through a 0.45 μm membrane, and stored at 4 °C for subsequent use. Determination of Ca, Li, Mg, Sr, Rb, Fe, Mn, Ni, Zn, Ba, As, Cr, Mo, Cu, Al, Hg, Be, Cd, Pb, Zr and Ta concentrations in lithium residue leachate by inductively coupled plasma mass spectrometry ICP-MS (NexION® 1000 ICP-MS, PerkinElmer Inc., Waltham, MA, USA).

### 2.2. Experimental Materials and Organisms

In this study, wild-type adult zebrafish of the AB strain were used (purchased from Nanjing Yishuli Hua Biotechnology Co., Ltd., Nanjing, China). Fish were maintained under a 14 h light/10 h dark photoperiod in water at pH 7.0, with the temperature maintained at 28.5 °C throughout the year. Zebrafish were fed freshly hatched brine shrimp three times daily (morning, noon, and evening). Before experimentation, sexually mature male and female zebrafish were transferred to breeding tanks at female-to-male ratios of 1:1 or 1:2, with transparent dividers used to separate the sexes. The dividers were removed the following morning to initiate spawning. Fertilised embryos were collected at approximately 11:00 A.M. using a Pasteur pipette and transferred to a temperature-controlled incubator set at 28.5 °C for subsequent cultivation.

### 2.3. Exposure Experiment of Leachate on Zebrafish Larvae

Two treatment groups were established using lithium slag leachate prepared at solid-to-liquid ratios of 1:10 and 1:1 (kg/L), with corresponding pH values of 7.5 and 8.2, respectively. Each treatment group consisted of six replicates, along with a parallel control group. Zebrafish embryos at 6 h post-fertilisation (hpf) were randomly allocated into culture dishes containing 150 embryos each and maintained at 28.5 °C in a temperature-controlled incubator. Exposure media were renewed daily, and dead embryos were promptly removed. Behavioural tests were performed on day 7 of exposure, after which zebrafish larvae were collected, immediately snap-frozen in liquid nitrogen, and stored at −80 °C for subsequent analysis.

### 2.4. Morphological Analysis

Following exposure to lithium slag leachate, morphological changes in the embryos were observed using a fluorescence microscope (Leica M205 FA, Leica Microsystems, Wetzlar, Germany) and a stereomicroscope (SZ680, Chongqing Aote Optical Instruments Co., Ltd., Chongqing, China), respectively. The parameters assessed included body length and weight after 7 days of exposure, hatching rates at 48, 60 and 72 h post-exposure, and the presence of pericardial oedema at 96 h post-exposure.

### 2.5. Determination of Metals in Zebrafish Larvae

Thirty zebrafish larvae per group (*n* = 5 replicates) were freeze-dried. The lyophilised samples were accurately weighed and transferred to microwave digestion vessels, to which 8 mL of nitric acid was added. After sealing, the samples were allowed to stand for 1 h. The sealed vessels were then placed into a polytetrafluoroethylene high-pressure digestion system for microwave-assisted digestion [27]. After cooling, the vessels were carefully opened to release residual gases. The inner lids were rinsed with a small volume of ultrapure water, and the digestion solution was transferred to a temperature-controlled hotplate or ultrasonic water bath for further processing. Finally, the digest was diluted to a final volume of 10 mL with ultrapure water and thoroughly mixed. Elemental concentrations were determined using ICP-MS.

### 2.6. Motion Analysis

Zebrafish embryos at 6 h post-fertilisation (hpf) were exposed to lithium slag leachate until 7 days post-fertilisation (dpf), with 20 larvae per group (*n* = 20). The method was adapted from the experimental protocol of Liu et al. [28] with appropriate modifications. After being rinsed twice with fish medium, larvae were individually transferred to wells of a 96-well plate, each containing an equal volume of fresh fish medium. Behavioural analysis was performed using the Labmaze v3.0 (ZS-Labmaze zeb, Beijing Zhongshi Dichuang Technology Development Co., Ltd., Beijing, China) system under alternating light–dark cycles and circadian rhythm-tracking conditions. Average velocity and total distance travelled were recorded and analysed. Three independent replicate experiments were performed.

### 2.7. Oxidative Stress Analysis

After 7 days of exposure, 30 zebrafish larvae were collected per centrifuge tube, with eight replicates per experimental group. Samples were homogenised in physiological saline at a mass-to-volume ratio of 1:9 on ice to obtain a 10% tissue homogenate. The homogenates were centrifuged at 2500 rpm for 10 min, and the supernatants were collected for subsequent analysis. Levels of total protein (TP), reactive oxygen species (ROS), superoxide dismutase (SOD), catalase (CAT), glutathione (GSH), and malondialdehyde (MDA) were determined using commercial assay kits (Nanjing Jiancheng Bioengineering Institute, Nanjing, China) according to the manufacturer’s instructions.

### 2.8. RNA Isolation, cDNA Library Construction, and Sequencing

To elucidate the molecular mechanisms underlying the effects of lithium slag leachate on zebrafish larvae, total RNA was extracted from pooled samples of 30 larvae from both control and exposed groups, using Trizol reagent from whole zebrafish larvae, with five biological replicates per group. Subsequently, the quality and integrity of these RNAs were assessed using an Agilent 5400 (Reagent Catalogue Number: DNF-471, Agilent Technologies, Santa Clara, CA, USA) system. Subsequently, RNA libraries were constructed and sequenced on the Illumina NovaSeq platform, generating 150 bp paired-end reads. Sequencing was performed by Novogene Co., Ltd. (Beijing, China), with differentially expressed genes identified using the criteria of *p* < 0.05 and |Log2FoldChange| > 1 [29]. Subsequently, GO enrichment analysis was performed on the differentially expressed genes using the clusterProfiler software (Version number: 3.8.1), with gene length bias corrected during the analysis; at the same time, KEGG pathway enrichment analysis was conducted using this software.

### 2.9. Fluorescent qPCR Validation of Transcriptomic Analysis

To validate the RNA sequencing results, qPCR was performed. At 7 dpf following exposure to lithium slag leachate, 30 zebrafish larvae were randomly sampled from each group, with four biological replicates per treatment. Individual larvae were placed in 1.5 mL RNase-free microcentrifuge tubes, with excess moisture carefully removed. The samples were immediately snap-frozen in liquid nitrogen and stored at −80 °C. Total RNA was extracted using a single-step TRIzol method. RNA concentration and purity were assessed to ensure suitability for downstream analysis, after which cDNA was synthesised using a commercial reverse transcription kit (Nanjing Jiancheng Bioengineering Institute, Nanjing, China). The qPCR amplification programme included an initial denaturation at 95 °C for 30 s, followed by 40 cycles of 95 °C for 5 s and 60 °C for 30 s. Transcript levels of neural and circadian rhythm-related genes were quantified, with β-actin used as the internal reference gene. Relative gene expression levels were calculated using the 2^−ΔΔCt^ method (Table S1).

### 2.10. Neurotransmitter Assay

At 7 dpf, 30 zebrafish larvae were randomly collected from each experimental group, with five biological replicates per group. Individual larvae were transferred to 1.5 mL microcentrifuge tubes, and excess moisture was carefully removed. These samples were then stored at −80 °C until analysis. Larvae were homogenised in ice-cold physiological saline at a mass-to-volume ratio of 1:9 to obtain a 10% tissue homogenate. The homogenates were centrifuged at 2500 rpm for 10 min, and the supernatants were collected for subsequent analysis. Levels of DA, 5-HT, gamma-aminobutyric acid (GABA), and norepinephrine (NE) were determined using ELISA kits (Nanjing Jiancheng Bioengineering Institute, Nanjing, China). Acetylcholine (ACh) content and activity were determined using a commercial assay kit from the same manufacturer. All measured values were normalised to total protein concentrations measured using the BCA method.

### 2.11. Data Analysis

All experimental data were statistically analysed using GraphPad Prism 10.0. Results are expressed as the mean ± standard deviation. Differences between the lithium slag leachate treatment group and the control group were assessed using one-way analysis of variance (ANOVA), followed by Dunnett’s multiple comparison test. All experiments included at least three independent replicates. Statistical significance was defined as * *p* < 0.05, ** *p* < 0.01, and *** *p* < 0.001.

## 3. Results

### 3.1. Analysis of Lithium Slag Leachate Composition

The physicochemical characteristics of the lithium slag leachate are summarised in Table 1. The leachate exhibited weak alkalinity, with pH values of 7.5 and 8.2 in the low- and high-concentration groups, respectively. Elemental analysis revealed the presence of multiple heavy metals, including iron, manganese, nickel, zinc, arsenic, chromium, copper, mercury, cadmium, and lead. In the high-concentration group, Fe showed the highest concentration among the heavy metals, followed by Mn and Ni. Among the metallic elements detected, calcium showed the highest concentration, reaching 490,000 μg/L in the high-concentration leachate group. Lithium concentrations were 17,000 μg/L and 98,000 μg/L in the low- and high-concentration groups, respectively. Compared with the control, Li levels increased by approximately 40,833-fold in the high-concentration group and 7083-fold in the low-concentration group. Similarly, Fe concentrations in the high- and low-concentration groups were approximately 143- and 81-fold higher than those in the control, respectively. According to the “Standards for drinking water quality” (GB 5749-2022) [30], concentrations of Fe, Mn, Ni, and As in the high-concentration group exceeded the permissible limits by factors of 10, 14, 6, and 4.7, respectively. These results indicate that lithium slag leachate contains a complex mixture of pollutants and may pose potential ecological risks.

### 3.2. Metal Accumulation and Concentration Changes in Zebrafish Larvae

**Figure 1:**
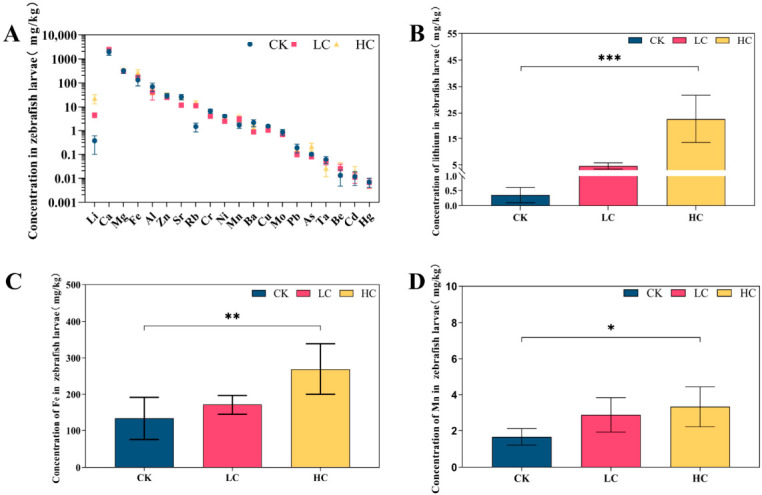
Metal accumulation in zebrafish larvae exposed to lithium slag leachate. (**A**) Elemental concentrations in zebrafish tissues. (**B**) Li concentration. (**C**) Fe concentration. (**D**) Mn concentration. Data are presented as mean ± SD (*n* = 5). * *p* < 0.05, ** *p* < 0.01, *** *p* < 0.001 compared with the control group. CK, control group; LC, low-concentration group; HC, high-concentration group.

To evaluate the accumulation of metals from lithium slag leachate, concentrations of multiple elements were measured in zebrafish larvae following 7 days of exposure (Figure 1A). Lithium levels increased to 13.35- and 65.78-fold of the control (CK) in the low- and high-concentration groups, respectively (Figure 1B). These results indicate that under high exposure conditions, lithium accumulation in leachate reaches elevated levels, consistent with the observed bioaccumulation pattern in zebrafish larvae. Furthermore, Fe and Mn concentrations in the high-concentration group were significantly elevated, reaching 2.01- and 2.00-fold of the control levels, respectively (Figure 1C,D).

### 3.3. Developmental Toxicity of Lithium Slag Leachate in Zebrafish Larvae

**Figure 2:**
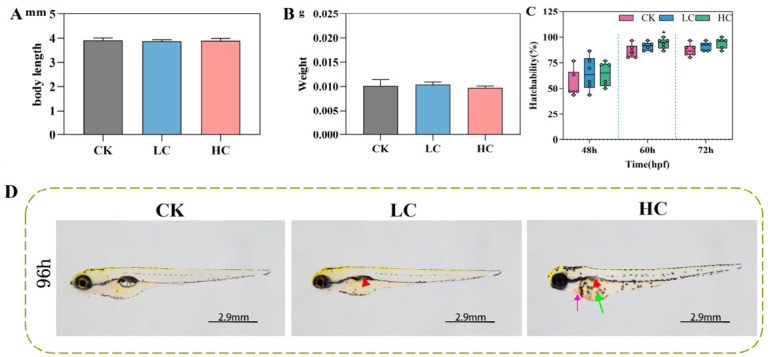
Developmental effects of lithium slag leachate on zebrafish larvae. (**A**) Body length of zebrafish larvae after 7 days of exposure (*n* = 15). (**B**) Body weight of zebrafish larvae after 7 days of exposure (*n* = 6). (**C**) Hatching rate of zebrafish embryos after 7 days of exposure (*n* = 6). (**D**) Representative morphological phenotypes of zebrafish larvae at 96 h post-fertilisation after leachate exposure. Red arrow: Juvenile fish swim bladder; Purple arrow: Juvenile fish heart; Green arrow: Juvenile fish yolk sac. Data are presented as mean ± SD. * *p* < 0.05. CK, control group; LC, low-concentration group; HC, high-concentration group.

Zebrafish are highly sensitive to environmental pollutants during early developmental stages [32]. To evaluate the developmental toxicity of lithium slag leachate, embryos were exposed to two leachate concentrations, and their morphological development was systematically evaluated. After 7 days of exposure, no significant differences in body length or weight were observed between the exposed and control groups (Figure 2A,B). Similarly, hatching rates at 48 and 72 hpf showed no significant variation between the exposed and control groups. However, at 60 hpf, embryos in the high-concentration group exhibited a significantly higher hatching rate (7.78% increase) compared with the control group (Figure 2C). Morphological examination at 96 hpf showed normal swim bladder inflation and no observable abnormalities, such as cardiac haemorrhage or yolk sac oedema, in control larvae. In contrast, larvae exposed to lithium slag leachate showed pronounced developmental defects, including uninflated swim bladders, yolk sac oedema, and cardiac haemorrhage. However, compared with the control group, the difference was not statistically significant (Figure 2D). Collectively, these findings indicate that lithium slag leachate induces significant developmental toxicity in zebrafish larvae.

### 3.4. Changes in Movement Behaviour

**Figure 3:**
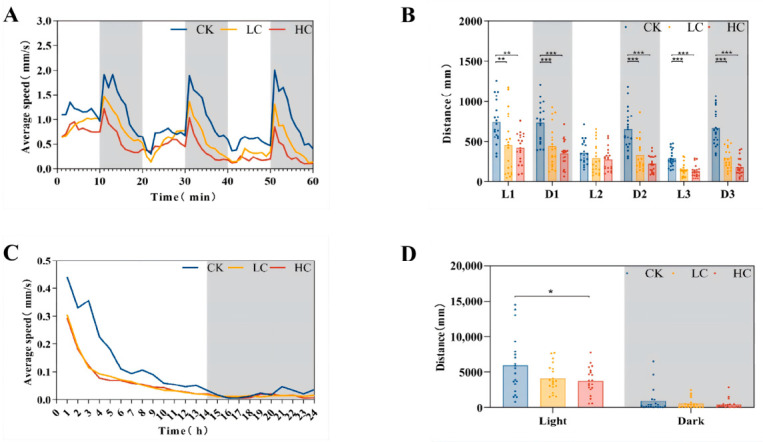
Effects of lithium slag leachate on swimming behaviour of zebrafish larvae after 7 days of exposure. (**A**) Average swimming speed under alternating light–dark cycles (*n* = 20). (**B**) Total distance travelled under alternating light–dark cycles (*n* = 20). (**C**) Average swimming speed during day–night periods (*n* = 20). (**D**) Total distance travelled during day–night periods (*n* = 20). Data are presented as mean ± SD. * *p* < 0.05, ** *p* < 0.01, *** *p* < 0.001. CK, control group; LC, low-concentration group; HC, high-concentration group.

Motor behaviour is a sensitive indicator of developmental integrity, reflecting the integrated functioning of the body’s various systems and its overall capacity for survival during the early stages of life [33]. In this study, zebrafish embryos at 6 hpf were exposed to lithium slag leachate for 7 days, and their locomotor activity was assessed under alternating light–dark cycles (10 min light/10 min dark) using an automated behavioural tracking system. The results showed that lithium slag leachate exposure significantly suppressed spontaneous locomotion in zebrafish larvae in a concentration-dependent manner. Both average swimming velocity and total distance travelled were markedly reduced compared with the control group. Although zebrafish normally exhibit higher activity during dark phases than light phases, larvae in the exposed groups showed markedly impaired movement during all three dark periods. Specifically, swimming distance decreased by 39.8% and 51.8% in the low- and high-concentration groups during the first dark phase, by 48.9% and 65.9% during the second dark phase, and by 56.4% and 72.6% during the third dark phase, respectively (Figure 3A,B). Circadian rhythm disruption is closely associated with a range of pathological conditions, including metabolic dysregulation, accelerated ageing, and neuropsychiatric disorders [34]. In this study, leachate exposure significantly disturbed normal circadian locomotor patterns, with larvae in the high-concentration group travelling only 0.63 times the distance of control larvae during light phases (Figure 3C,D).

### 3.5. Changes in Oxidative Stress-Related Markers

**Figure 4:**
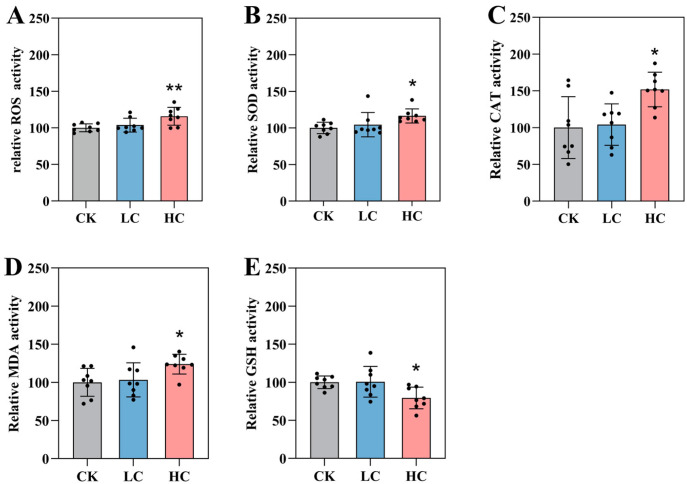
Oxidative stress indicators in zebrafish larvae exposed to lithium slag leachate. (**A**) Reactive oxygen species (ROS) levels. (**B**) Superoxide dismutase (SOD) activity. (**C**) Catalase (CAT) activity. (**D**) Malondialdehyde (MDA) content. (**E**) Glutathione (GSH) content. Data are presented as mean ± SD (*n* = 8). * *p* < 0.05, ** *p* < 0.01. CK, control group; LC, low-concentration group; HC, high-concentration group.

After 7 days of exposure to lithium slag leachate, zebrafish larvae were assessed for oxidative stress by measuring ROS and key antioxidant parameters, including SOD, CAT, MDA, and GSH. Oxidative stress occurs when ROS generation exceeds the capacity of the antioxidant defence system. As shown in Figure 4A, ROS levels in the high-concentration group significantly increased to 115.74 nmol/mg compared with the control, whereas the low-concentration group showed no significant change, indicating limited ROS induction at lower exposure levels. In the high-concentration group, activities of the antioxidant enzymes SOD and CAT, as well as the lipid peroxidation marker MDA activities, were significantly elevated, reaching 116.40, 151.82, and 123.90 U/mg protein, respectively (Figure 4B–D). In contrast, the activity of the non-enzymatic antioxidant GSH was significantly reduced to 79.34 U/mg protein (0.79-fold of the control), while no significant change was observed in the low-concentration group (Figure 4E).

### 3.6. Lithium Slag Leachate Alters the Global Transcriptome

**Figure 5:**
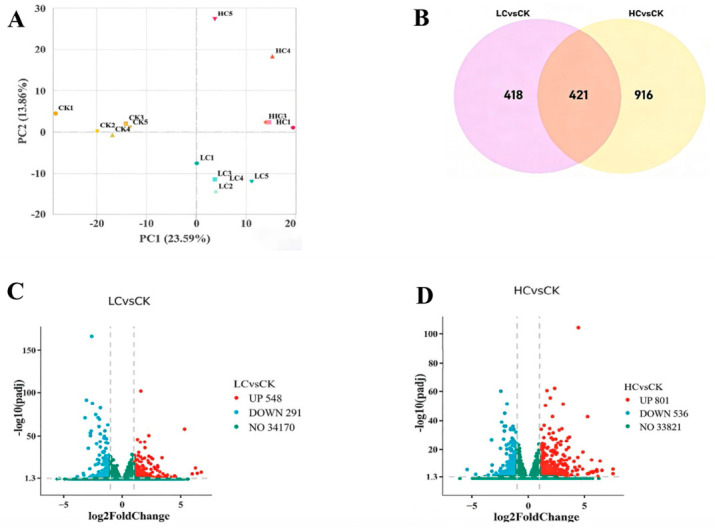
Analysis of gene expression levels and the number of differentially expressed genes. (**A**) Principal component analysis (PCA). (**B**) Venn diagram of differentially expressed genes (DEGs). (**C**) DEGs between the control and low-concentration groups. (**D**) DEGs between the control and high-concentration groups.

RNA sequencing was performed on zebrafish larvae exposed to low and high concentrations of lithium slag leachate and on control larvae. Principal component analysis (PCA) showed clear separation among the control, low-concentration, and high-concentration groups along the first two principal components, indicating distinct transcriptomic profiles and a concentration-dependent response to leachate exposure (Figure 5A). Differential expression analysis identified 839 DEGs in the low-concentration group (548 upregulated and 291 downregulated) and 1337 DEGs in the high-concentration group (801 upregulated and 536 downregulated), with 421 genes shared between the two exposure groups (Figure 5B–D).

**Figure 6:**
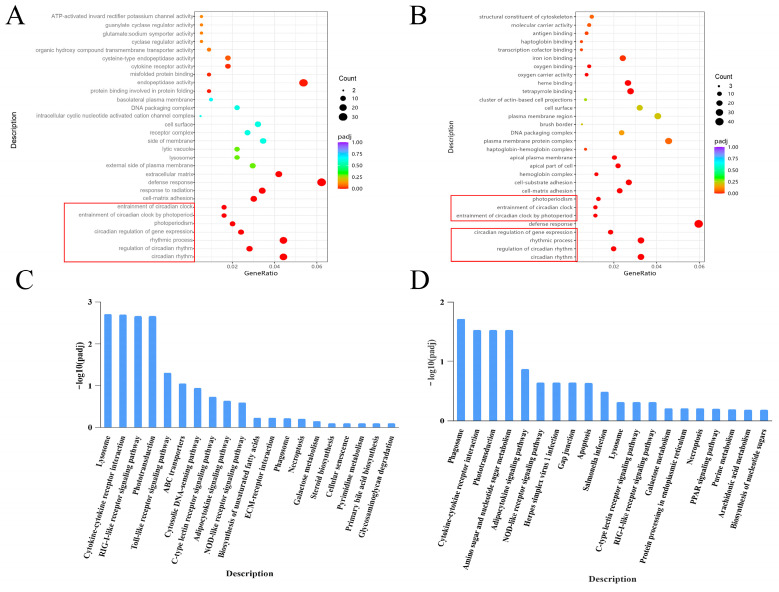
Differential gene enrichment analysis. (**A**) Gene ontology (GO) functional enrichment analysis for the low-concentration group. The red boxes represent pathways related to the circadian rhythm. (**B**) GO functional enrichment analysis for the high-concentration group. The red boxes represent pathways related to the circadian rhythm. (**C**) Kyoto Encyclopaedia of Genes and Genomes (KEGG) pathway enrichment analysis for the low-concentration group. (**D**) KEGG pathway enrichment analysis for the high-concentration group. CK, control group; LC, low-concentration group; HC, high-concentration group.

GO enrichment analysis revealed significant disruption of biological processes associated with circadian rhythm, regulation of rhythmic processes, and response to light stimuli, suggesting disruption of the zebrafish circadian clock. Notably, seven core circadian rhythm genes—*per1a*, *per1b*, *per2*, *per3*, *nr1d1*, *cry2*, and *cry5*—were consistently downregulated (Figure 6A,B). The criteria for identifying differentially expressed genes were |log_2_FC| ≥ 1, with the FDR calculated using the Benjamini–Hochberg method for multiple comparison correction. KEGG pathway analysis showed that the low-concentration group was primarily enriched in immune-related pathways, including “cytokine–cytokine receptor interaction” and “RIG-I-like receptor signalling,” indicating early immune activation. In contrast, the high-concentration group exhibited stronger enrichment of defence-related pathways such as “phagosome” and “Salmonella infection,” reflecting an intensified immune response at higher exposure levels (Figure 6C,D).

### 3.7. Validation of Circadian Rhythm-Related Gene Expression

**Figure 7:**
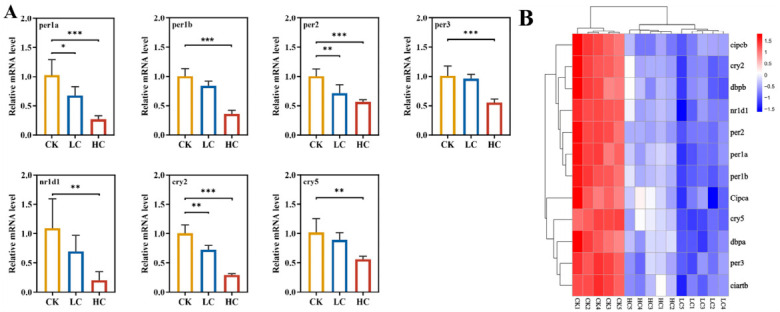
Gene expression changes following exposure to lithium slag leachate. (**A**) Expression changes in genes related to circadian rhythm and light input pathways. (**B**) Heatmap of 13 common differentially expressed genes across CK, LC, and HC groups. Data represent mean ± SD (*n* = 4). * *p* < 0.05, ** *p* < 0.01, *** *p* < 0.001. CK, control group; LC, low-concentration group; HC, high-concentration group.

To further assess the effect of lithium slag leachate on the expression of circadian rhythm-related genes in zebrafish, qRT-PCR was used to measure the transcription levels of key circadian rhythm genes. Validation confirmed significant alterations in seven circadian-associated genes (*per1a*, *per1b*, *per2*, *per3*, *nr1d1*, *cry2*, and *cry5*) (Figure 7A). The circadian rhythm represents a fundamental 24 h oscillation regulating essential physiological processes, including sleep–wake cycles, metabolism, thermoregulation, hormone secretion, and immune function. Core clock genes (*per1a*, *per1b*, *per2*, *per3*, and *nr1d1*) form a transcriptional–translational negative feedback loop that generates and maintains endogenous ~24 h rhythms through auto-repressive mechanisms. In the high-concentration group, all five core genes were significantly downregulated. Additionally, *cry2* and *cry5* primarily function as photoreceptors that detect environmental light–dark cues and synchronise the internal clock with external conditions. Expression of *cry5* decreased by 54.96% in the high-concentration group relative to controls, while *cry2* showed reductions of 27.46% and 70.50% in the low- and high-concentration groups, respectively (Figure 7A).

GO pathway analysis of 13 circadian rhythm-related DEGs revealed that lithium slag leachate exposure disrupts circadian regulation. The altered expression patterns of these genes suggest that leachate components interfere with normal circadian function, leading to dysregulated physiological rhythms (Figure 7B).

### 3.8. Neurotransmitters and Related Gene Analysis

**Figure 8:**
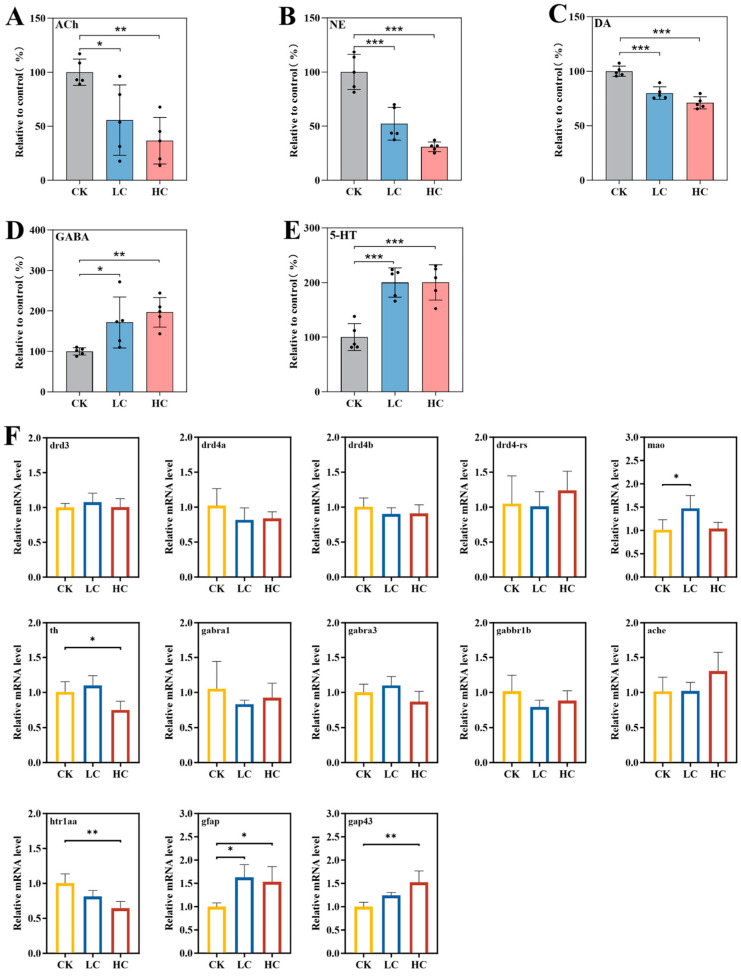
Neurotransmitter levels and related gene expression following exposure to lithium slag leachate. (**A**) Acetylcholine (ACh) content (*n* = 5). (**B**) Norepinephrine (NE) content (*n* = 5). (**C**) Dopamine (DA) content (*n* = 5). (**D**) Gamma-aminobutyric acid (GABA) content (*n* = 5). (**E**) Serotonin (5-HT) content (*n* = 5). (**F**) Real-time quantitative PCR (qPCR) validation of mRNA expression levels for neurosystem genes (*drd3*, *drd4a*, *drd4b*, *drd4-rs*, *mao*, *th*, *gabra1*, *gabra3*, *gabbr1b*, *ache*, *htr1aa*, *gfap*, and *gap43*) after lithium slag leachate exposure (*n* = 4). Data are presented as mean ± SD, * *p* < 0.05, ** *p* < 0.01, *** *p* < 0.001. CK, control group; LC, low-concentration group; HC, high-concentration group.

To explore neurochemical mechanisms underlying the behavioural alterations induced by lithium slag leachate, key neurotransmitters—including ACh, NE, DA, GABA, and 5-HT—were quantitatively analysed. After 7 days of exposure, significant reductions in ACh, NE, and DA levels were observed, accompanied by marked increases in GABA and 5-HT levels. The decreases in ACh, NE, and DA showed a clear concentration-dependent trend. ACh, a classical neuromodulator, regulates neuronal excitability and modulates responses to both internal and external stimuli [35]. Compared with the control group, ACh content decreased by 44.34% and 63.43% in the low- and high-concentration groups, respectively (Figure 8A). NE, which functions in the autonomic nervous system, regulates multiple neurophysiological processes, including cognition, learning, memory, arousal, and reward mechanisms [36,37]. Its levels were significantly reduced by 47.74% and 69.11% in the low- and high-concentration groups, respectively (Figure 8B). DA, an essential monoaminergic neurotransmitter, is involved in reward processing, motor control, and memory formation in vertebrates, including teleost fish [38,39]. Its levels decreased by 20.05% and 29.00% in the low- and high-concentration groups, respectively (Figure 8C). GABA and 5-HT act as primary inhibitory neurotransmitters, modulating neuronal and postsynaptic activity [17,40]. GABA levels increased significantly in a concentration-dependent manner, reaching 1.72- and 1.96-fold of control values in the low- and high-concentration groups, respectively (Figure 8D). Similarly, 5-HT levels showed a marked upward trend, with the high-concentration group exhibiting a twofold increase relative to controls (Figure 8E).

Transcriptional analysis revealed significant upregulation of neuronal development-related genes (*gap43* and *gfap*) in the high-concentration group. The 5-HT receptor gene *htr1aa* was significantly downregulated relative to controls. In contrast, DA receptor genes (*drd3*, *drd4a*, *drd4b*, and *drd4-rs*), GABA receptor genes (*gabra1*, *gabra3*, and *gabbr1b*), and the acetylcholinesterase-encoding gene (*ache*) showed no significant alterations. Monoamine oxidase (*mao*), which catalyses the degradation of biogenic amine neurotransmitters, including 5-HT, DA, and NE, was significantly upregulated in the low-concentration group, reaching 1.45-fold of control levels. Conversely, tyrosine hydroxylase (*th*), the rate-limiting enzyme in catecholamine synthesis, was significantly downregulated to 0.75-fold of control in the high-concentration group (Figure 8F).

## 4. Discussion

Lithium slag, a by-product generated during lithium carbonate production, is frequently disposed of in landfills. This practice, however, poses potential environmental risks, as contaminants may leach into surrounding water bodies, soils, and surrounding ecosystems. Our analysis shows that lithium slag leachate contains a variety of metallic elements, including heavy metals such as Fe, Mn, Ni, Zn, As, Cr, Cu, Hg, Cd, and Pb. Among metallic elements, Li and Ca are predominant. Therefore, using zebrafish as a model organism to investigate the effects of lithium slag leachate—particularly its disruption of neural function and circadian rhythms—provides valuable insights for the ecological risk assessment of lithium slag and science-based environmental management. In natural aquatic systems, the co-occurrence of multiple pollutants can produce complex interactions and combined toxic effects. Even low-level exposure to individual contaminants may amplify ecological and health risks through cumulative or synergistic actions, highlighting the need for further research on mixture toxicity [41,42].

This study first evaluated the developmental toxicity of lithium slag leachate. With increasing exposure concentrations, zebrafish larvae exhibited characteristic malformations, including yolk sac oedema, cardiac haemorrhage, and uninflated swim bladders, indicating clear developmental toxicity. Behaviour serves as an integrated physiological response to environmental stimuli, and behavioural alterations are recognised as sensitive indicators of pollutant exposure and bioaccumulation [43]. In zebrafish neuropharmacology, behavioural analysis provides a cost-effective strategy, with observed changes directly reflecting nervous system impairments. Consistent with previous reports of locomotor deficits following heavy metal exposure [44,45]. Our results showed that a 7-day exposure to lithium slag leachate significantly reduced both average velocity and total distance travelled in zebrafish larvae, confirming compromised locomotor capacity and neurotoxic potential.

ROS, naturally generated during mitochondrial metabolism, are typically maintained at physiological levels by endogenous antioxidant systems. Under chemical stress, however, excessive ROS accumulation can damage lipids, proteins, and nucleic acids [46]. The cellular antioxidant defence system—comprising enzymes such as SOD and CAT, along with reduced GSH—neutralises excess ROS and maintains redox homeostasis [47]. In our study, exposure to lithium slag leachate significantly increased ROS levels in the high-concentration group. Concurrently, key antioxidant parameters were markedly altered: SOD and CAT activities increased significantly, while GSH levels declined. Similarly, combined exposure to arsenic (As) and chromium (Cr) induced oxidative stress in zebrafish, manifested by elevated levels of reactive oxygen species, reduced glutathione levels and increased lipid peroxidation [48]. MDA, a well-established lipid peroxidation biomarker indicative of oxidative membrane damage [49,50], was significantly higher in the high-concentration group than in controls. Together, these findings indicate that lithium slag leachate compromises antioxidant defence capacity and induces oxidative stress in zebrafish larvae.

Transcriptomic analysis revealed that exposure to lithium slag leachate at varying concentrations significantly altered gene expression in zebrafish larvae. These changes were accompanied by disruptions in circadian rhythms and marked downregulation of multiple core circadian clock and melanopsin-related genes. These genes are key components of the circadian transcriptional–translational feedback loop, in which rhythmic expression is initiated by the CLOCK/BMAL1 heterodimer and regulated through negative feedback by core clock genes [51,52]. The observed downregulation thus indicates a disruption of circadian regulation. qPCR analysis confirmed significant downregulation of core circadian genes—including *per1a*, *per1b*, *per2*, *per3*, *nr1d1*, *cry2*, and *cry5*—following lithium slag leachate exposure, indicating interference with the zebrafish circadian clock. Dysregulation of *cry* and *per* genes is known to induce various adverse physiological effects. For example, prednisolone causes circadian phase delays in zebrafish by aberrantly upregulating *per* and *cry* genes, suppressing melatonin synthesis, and promoting sleep disturbances [53]. Studies in zebrafish, goldfish, and related species also show that ocular *cry* genes exhibit distinct expression patterns under different photoperiods, with transcript levels oscillating in synchrony with dawn, dusk, or other diurnal phases, reflecting sophisticated, light-entrained oscillatory mechanisms in the eye [54].

Neurotransmitters are endogenous signalling molecules that mediate synaptic communication and play vital roles in numerous physiological processes. Dysregulation of neurotransmitter levels is implicated not only in various pathological conditions [55,56] but also serves as a sensitive indicator of neurotoxic exposure [57,58]. Previous studies have shown that organic environmental contaminants—including triphenyl phosphate, diazinon, and perfluorooctanoic acid—disrupt synaptic function and induce neurotoxicity in zebrafish [28,59,60]. In the present study, lithium slag leachate exposure significantly disrupted neurotransmitter homeostasis, evidenced by reduced levels of ACh, NE, and DA, alongside elevated concentrations of GABA and 5-HT. These changes indicate interference with neurotransmitter synthesis, release, or metabolic pathways. Similar disruptions in ACh and DA have been reported in zebrafish exposed to fenpyroximate [61]. Moreover, exposure to heavy metals is known to induce neurotransmitter imbalances and behavioural deficits across species [62,63,64], a finding supported by the present study. Accordingly, neurotransmitter dysregulation represents a plausible mechanism underlying the altered neurobehavioural responses observed in zebrafish, potentially resulting from metal ions interfering with neurotransmitter release and ligand–receptor interactions. Consistent with these findings, gene expression analysis revealed significant changes in neural-related transcripts. For instance, glial fibrillary acidic protein (*gfap*)—an intermediate filament protein highly expressed in astrocytes—is essential for maintaining cytoskeletal integrity and mediating neuroinflammatory responses [65]. Similarly, *Gap43*, an axon growth-associated protein, plays a critical role in neural development and regenerative processes following central nervous system injury in zebrafish and other teleosts, contributing to the maintenance of neuronal growth and plasticity [66]. The upregulation of *gfap* and *gap*43 under high-concentration exposure likely reflects a compensatory or stress-induced neural response to chemical challenge. Tyrosine hydroxylase (*th*), first identified at the National Institutes of Health (NIH) in 1964, is a tetrahydrobiopterin (BH_4_)-dependent monooxygenase that catalyses the rate-limiting step in the biosynthesis of catecholamines, including DA, NE, and epinephrine [67]. Our results showed significant downregulation of *th* gene expression in the high-concentration group, consistent with the observed reductions in DA and NE levels. Given its central role in catecholamine synthesis, the suppression of *th* would directly limit the production of these neurotransmitters. In the central nervous system, *mao* is responsible for the degradation of monoamine neurotransmitters [68,69]. Although *mao* expression was upregulated at low exposure concentrations—potentially enhancing neurotransmitter turnover—the overall outcome was a significant reduction in NE and DA levels. The elevated 5-HT levels may reflect a compensatory adjustment to maintain excitatory–inhibitory balance. Notably, DA receptor genes (*drd3*, *drd4a*, *drd4b*, and *drd4-rs*), GABA receptor genes (*gabra1*, *gabra3*, and *gabrb1b*), and most neurotransmitter-related enzyme genes did not show significant differential expression; the marked reductions in ACh and DA, coupled with increased GABA levels, suggest that neurotransmitter imbalances arise primarily from dysregulation of synthesis, release, or transport mechanisms rather than from direct alterations in receptor or enzyme gene expression.

## 5. Conclusions

Lithium slag can enter aquatic systems through surface runoff during precipitation events, facilitating the transport of hazardous constituents and posing risks to water quality. Its persistence and accumulation in the environment represent potential health threats to both aquatic organisms and humans. Our study shows that exposure to lithium slag leachate induces developmental malformations in zebrafish larvae, accompanied by behavioural alterations, oxidative stress, circadian rhythm disruption, and neurotransmitter imbalance. These findings provide a scientific basis for an in-depth understanding of the toxicity mechanisms of complex metal mixtures in lithium slag. In the future, long-term ecological monitoring should be conducted at representative lithium slag storage sites. By combining multi-dimensional analyses of water, sediment and biological samples, the migration, enrichment and bioaccumulation of pollutants from lithium slag within regional ecosystems should be investigated, thereby enabling a more accurate assessment of the ecological risks they pose to the structure and function of aquatic communities.

## Figures and Tables

**Table 1:** Water quality characteristics of lithium slag leachate.

Element	Mean Concentration and Standard Deviation (μg/L)			Environmental Quality Standards for Surface Water (GB 3838-2002): Class V Water Standard (μg/L) [31]	Standards for Drinking Water Quality (GB 5749-2022): Quality Limit (μg/L)
	CK	LC	HC		
Ca	680 ± 10	120,000 ± 11,000	490,000 ± 1700	/	/
Li	2.4 ± 0.17	17,000 ± 1700	98,000 ± 21,000	/	/
Mg	8800 ± 73	3800 ± 740	14,000 ± 4600	/	/
Sr	43 ± 0.63	4600 ± 930	7200 ± 300	/	/
Rb	0.24 ± 0.013	460 ± 20	5000 ± 700	/	/
Fe	21 ± 0.58	1700 ± 200	3000 ± 590	/	300
Mn	0.69 ± 0.014	1800 ± 20	1400 ± 90	/	100
Ni	0.93 ± 0.011	91 ± 6.7	120 ± 1.9	/	20
Zn	3.1 ± 0.082	27 ± 7.5	82 ± 16	≤2000	1000
Ba	4.6 ± 0.10	64 ± 5.3	83 ± 1.5	/	700
As	0.060 ± 0.016	7.8 ± 0.28	47 ± 7.9	≤100	10
Cr	0.24 ± 0.013	5.6 ± 0.90	16 ± 3.8	≤100	50
Mo	0.84 ± 0.011	5.9 ± 0.74	19 ± 1.8	/	70
Cu	0.78 ± 0.021	0.64 ± 0.36	1.4 ± 0.25	≤1000	1000
Al	1.0 ± 0.028	3.1 ± 1.0	1.1 ± 0.053	/	200
Hg	0.0031 ± 0.00024	0.11 ± 0.072	0.47 ± 0.10	≤1	1
Be	0.0042 ± 0.0011	0.12 ± 0.022	0.41 ± 0.041	/	2
Cd	0.0072 ± 0.00020	0.20 ± 0.028	0.29 ± 0.12	≤10	5
Pb	0.045 ± 0.0022	0.023 ± 0.0082	0.15 ± 0.039	≤100	10
Zr	0.015 ± 0.0014	0.058 ± 0.0093	0.15 ± 0.0074	/	/
Ta	0.011 ± 0.0011	0.0072 ± 0.0011	0.038 ± 0.017	/	/

## Data Availability

The original contributions presented in this study are included in the article/Supplementary Materials. Further inquiries can be directed to the corresponding authors.

## Supplementary Materials

The following supporting information can be downloaded at https://www.mdpi.com/article/10.3390/toxics14040345/s1, Table S1. Primers used for qRT-PCR validation [28,34,70,71,72,73,74,75,76,77,78,79].
