# Supplementary material for: Multi-Metal Leachate from Lithium Slag Induces Oxidative Stress, Circadian Disruption, and Neurobehavioural Toxicity in Zebrafish Larvae
Source: Toxics. 2026 Apr 20;14(4):345. doi: 10.3390/toxics14040345 (PMC13120298; doi:10.3390/toxics14040345)
Supplement: Supplementary file 1 [file toxics-14-00345-s001.zip › toxics-4240747-supplementary.pdf]

Multi-metal leachate from lithium slag induces oxidative stress, circadian disruption, and neurobehavioural toxicity in zebrafish larvae

Xueping Huang[1], Shengping Zhang[1], Yu Liu[2], Shuai Liu[3], Qiyu Wang[3], Nannan Wan[2], Shanghaojun Lu[1], Yongming Wu[2*], Miao Zhang[3*]

[1] School of Civil Engineering and Architectural Engineering, Jiangxi University of Water Resources and Electric Power, Nanchang 330099, China.

[2] Institute of Resources and Environment, Jiangxi Academy of Sciences, Nanchang 330096, China.

[3] Institute of Medicine and Health, Jiangxi Academy of Sciences, Nanchang 330096, China.

Table S1. Primers used for qRT-PCR validation.

Nomenclature	Primer type	Primer sequence (5′–3′)	References
β-actin	forward	CGAGCAGGAGATGGGAACC	[70]
	reverse	CAACGGAAACGCTCATTGC	
per1a	forward	ATGCGTGCAAGAAGTGGTG	[71]
	reverse	ACGTCCTCATTTAGCGGACTC	
per2	forward	CTCTGGACGGCAGTGAGAAT	
	reverse	CACAGCACCTTCTGGATGTC	
nr1d1	forward	ACATCCCAGGGTTTAGCACG	
	reverse	GTTCGGAGAGCCCGGATTAG	
per1b	forward	AGGAAGGCTGACAGATGATGAATG	[34]
	reverse	CCAGAGTGGGCTAAAGCGAAGTA	
per3	forward	GTTCTGGCGGAGTAATGGAG	
	reverse	TGACGACGTTTTACTGGTGC	
cry2	forward	AGAGTAAGACCCTCAGAGACCCC	[72]
	reverse	CGTTTCTGTCGGATGGTTCGGAG	
cry5	forward	CGGCATTAATCGATGGAGAT	[73]
	reverse	GCTTTGGGAGAACCTCTGTG	
drd3	forward	TGGTGAATGGCTCTGGAATGAC	[74]
	reverse	AGTGAGGCGACCAACAGATCAG	
drd4a	forward	ATCATGCTGCTCCTGTACTGCG	
	reverse	GATACGCCAACATGGACACAGG	
drd4b	forward	AACGTGCTCGTGTGCCTTAGTG	
	reverse	TCCACACTGATTGCGCACAG	

drd4-rs	forward	ATGCTCTGCACTGCCTCAAT	
	reverse	CCCAGTCGCAAAGACAGACT	
gabra1	forward	AGCGCAAGAAACTCTCTCCC	[28]
	reverse	GGTCTCTGGCAATGTTCGGA	
gabbr1b	forward	GGCCGTCAAGAACCTCAAGA	
	reverse	ACCAGTTGTCAGCGTACCAG	
mao	forward	GCAGTCAGAGCCCGAATC	
	reverse	CACACCCATAAACTTGAGGAATC	[75]
htr1aa	forward	AGAGCAGCGAGGTGAC	
	reverse	GAGCCGATGATTTGGTAAC	
th	forward	GCTCTCAGCACGCGATTTTT	[76]
	reverse	TCATGGACGCAATCCGGTTC	
gabra3	forward	GCTGAAGTTCGGGAGCTATG	[77]
	reverse	GGAGCTGATGGTCTCTTTGC	
ache	forward	CCCTCCAGTGGGTACAAGAA	
	reverse	GGGCCTCATCAAAGGTAACA	[78]
gfap	forward	GGATGCAGCCAATCGTAAT	
	reverse	TTCCAGGTCACAGGTCAG	
gap43	forward	CAGCCGACGTGCCTGAA	[79]
	reverse	GGATTCCTCAGCAGCGTCTG	

References

28. Liu, S.; Qiu, W.; Li, R.; Chen, B.; Wu, X.; Magnuson, J.T.; Xu, B.; Luo, S.; Xu, E.G.; Zheng, C. Perfluorononanoic Acid Induces Neurotoxicity via Synaptogenesis Signaling in Zebrafish. Environ Sci Technol. 2023, 57(9), 3783-3793.https://doi.brg/10.1021/acs.est.2c06739

34. Yang, Y.; Dong, F.; Liu, X.; Xu, J.; Wu, X.; Zheng, Y. Flutolanil affects circadian rhythm in zebrafish (Danio rerio) by disrupting the positive regulators. Chemosphere. 2019, 228, 649-655.https://doi.org/10.1016/j.chemosphere.2019.04.207

70. Tu, W.; Martínez, R.; Navarro-Martin, L.; Kostyniuk, D.J.; Hum, C.; Huang, J.; Deng, M.; Jin, Y.; Chan, H.M.; Mennigen, J.A. Bioconcentration and Metabolic Effects of Emerging PFOS Alternatives in Developing Zebrafish. Environ Sci Technol. 2019, 53(22), 13427-13439.https://doi.brg/10.1021/acs.est.9b03820

71. Ricarte, M.; Prats, E.; Montemurro, N.; Bedrossiantz, J.; Bellot, M.; Gómez-Canela, C.; Raldúa, D. Environmental concentrations of tire rubber-derived 6PPD-quinone alter CNS function in zebrafish larvae. Science of The Total Environment. 2023, 896, 165240.https://doi.org/10.1016/j.scitotenv.2023.165240

72. Zhou, Z.; Peng, X.; Chen, J.; Wu, X.; Wang, Y.; Hong, Y. Identification of zebrafish magnetoreceptor and cryptochrome homologs. Science China Life Sciences. 2016, 59(12), 1324-1331

73. Shi, W.-J.; Jiang, Y.-X.; Ma, D.-D.; Huang, G.-Y.; Xie, L.; Chen, H.-X.; Huang, M.-Z.; Ying, G.-G. Dydrogesterone affects the transcription of genes in visual cycle and circadian rhythm network in the eye of zebrafish. Ecotoxicology and Environmental Safety. 2019, 183, 109556.https://doi.org/10.1016/j.ecoenv.2019.109556

74. Hosseini, P.; Mirsadeghi, S.; Rahmani, S.; Izadi, A.; Rezaei, M.; Ghodsi, Z.; Rahimi-Movaghar, V.; Kiani, S. Dopamine receptors gene expression pattern and locomotor improvement differ between female and male zebrafish during spinal cord auto repair. Zebrafish. 2022, 19(4), 137-147

75. Attaran, A.; Salahinejad, A.; Crane, A.L.; Niyogi, S.; Chivers, D.P. Chronic exposure to dietary selenomethionine dysregulates the genes involved in serotonergic neurotransmission and alters social and antipredator behaviours in zebrafish (Danio rerio). Environ Pollut. 2019, 246, 837-844.https://doi.brg/10.1016/j.envpol.2018.12.090

76. Wang, J.; Gao, N.; Bian, Z.; Wang, Z.; Xun, Z.; Wu, L. The effects of DBP and BPA on early development, locomotor behaviour and the nervous system in zebrafish. *Acta Ecologica* **2025**, *45*, 1748–1762. https://doi.org/10.20103/j.stxb.202403100484.

77. Reyes-Nava, N.G.; Paz, D.; Pinales, B.E.; Perez, I.; Gil, C.B.; Gonzales, A.V.; Grajeda, B.I.;Estevao, I.L.;Ellis, C.C.;Castro, V.L.;Quintana, A.M. Characterization of the zebrafish gabra1(sa43718/sa43718) germline loss of function allele confirms a function for Gabra1 in motility and nervous system development. Differentiation. 2024, 138, 100790.https://doi.brg/10.1016/j.diff.2024.100790

78. Zhu, X.Y.; Wu, Y.Y.; Xia, B.; Dai, M.Z.; Huang, Y.F.; Yang, H.; Li, C.Q.; Li, P. Fenobucarb-induced developmental neurotoxicity and mechanisms in zebrafish. Neurotoxicology. 2020, 79, 11-19.https://doi.brg/10.1016/j.neuro.2020.03.013

79. Zeng, F.; Jin, M.; Shi, R.; Zhang, X.; Li, N. A Study on the Neurodevelopmental Toxicity and Parkinson's-like Symptoms Induced by Exposure to Nano-Aluminium Oxide in Zebrafish. *Environ. Occup. Med.* **2024**, *41*, 814–821.
